# Target Temperature Management Effect on the Clinical Outcome of Patients with Out-of-Hospital Cardiac Arrest Treated with Extracorporeal Cardiopulmonary Resuscitation: A Nationwide Observational Study

**DOI:** 10.3390/jpm14020185

**Published:** 2024-02-07

**Authors:** Jae-Hee Kim, Jae-Guk Kim, Gu-Hyun Kang, Yong-Soo Jang, Wonhee Kim, Hyun-Young Choi, Yoonje Lee, Chiwon Ahn

**Affiliations:** 1 Department of Emergency Medicine, Kangnam Sacred Heart Hospital, Hallym University College of Medicine, 1, Singil-ro, Yeongdeungpo-gu, Seoul 07441, Republic of Korea; jhkim6612@naver.com (J.-H.K.); emkang@hallym.or.kr (G.-H.K.); amicoys@hallym.or.kr (Y.-S.J.); wonsee02@hallym.or.kr (W.K.); chy6049@naver.com (H.-Y.C.); yong0831@naver.com (Y.L.); 2 Department of Emergency Medicine, College of Medicine, Chung-Ang University, Seoul 06974, Republic of Korea; cahn@cau.ac.kr

**Keywords:** extracorporeal cardiopulmonary resuscitation, targeted temperature management, cardiac arrest, survival, neurological outcome

## Abstract

This study aimed to investigate whether targeted temperature management (TTM) could enhance outcomes in patients with out-of-hospital cardiac arrest (OHCA) treated with extracorporeal cardiopulmonary resuscitation (ECPR) for refractory cardiac arrest. Using a nationwide OHCA registry, adult patients with witnessed OHCA of presumed cardiac origin who underwent ECPR at the emergency department between 2008 and 2021 were included. We examined the effect of ECPR with TTM on survival and neurological outcomes at hospital discharge using propensity score matching and multivariable logistic regression compared with patients treated with ECPR without TTM. Odds ratios and 95% confidence intervals were determined. A total of 399 ECPR cases were analyzed among 380,239 patients with OHCA. Of these, 330 underwent ECPR without TTM and 69 with TTM. After propensity score matching, 69 matched pairs of patients were included in the analysis. No significant differences in survival and good neurological outcomes between the two groups were observed. In the multivariable logistic regression, no significant differences were observed in survival and neurological outcomes between ECPR with and without TTM. Among the patients who underwent ECPR after OHCA, ECPR with TTM did not improve outcomes compared with ECPR without TTM.

## 1. Introduction

Out-of-hospital cardiac arrest (OHCA) remains a major global health concern with poor survival rates and significant neurological sequelae among survivors [1,2,3,4]. Extracorporeal cardiopulmonary resuscitation (ECPR) has emerged as a viable treatment option for patients with OHCA, with the potential to enhance survival and neurological outcomes [1,5]. However, optimizing patient management during and after ECPR is crucial to enhance their chances of recovery [6]. 

One area of active investigation is targeted temperature management (TTM), a therapeutic approach involving the deliberate induction and maintenance of hypothermia or controlled normothermia following the return of spontaneous circulation (ROSC) [7,8]. TTM has demonstrated benefits in patients post-cardiac arrest following conventional cardiopulmonary resuscitation, with favorable outcomes observed in terms of reduced neurological injury and improved survival [9]. However, the role of TTM in the context of ECPR, which provides advantages such as prolonged therapeutic time windows and expedited cooling, remains uncertain and requires further exploration [10,11,12]. 

Recent studies have reported contradictory results regarding the effectiveness of TTM after ECPR [10,11,12,13]. There is a paucity of comprehensive evidence to guide clinical practice and establish the actual impact of TTM on ECPR outcomes. Therefore, using nationwide data, our study examined the impact of combining ECPR with TTM on patient outcomes of OHCA as compared to the application of ECPR in the absence of TTM.

## 2. Methods

### 2.1. Study Design and Settings

This study, characterized by its retrospective observational nature, used data derived from a comprehensive, population-based dataset. This dataset was sourced from the Out-of-Hospital Cardiac Arrest Surveillance (OHCAS) database, maintained by the Korean Centers for Disease Control and Prevention (KCDC). The time frame for data collection spanned from January 2008 to December 2021.

The OHCAS initiative encompasses 17 provinces across South Korea, representing a population of approximately 50 million individuals, and offers comprehensive patient information for analysis. The conduct of this study was granted ethical clearance by the Institutional Review Board (IRB) of Kangnam Sacred Heart Hospital (IRB No. 2023-09-010) in the year 2023. Given the retrospective methodology of this research and the use of de-identified clinical data, the IRB accorded a waiver for the necessity of obtaining informed consent.

The OHCAS provides a comprehensive analysis at a national level of the pre-hospital characteristics of OHCA patients, including the presumed cause of cardiac arrest, incidents of witnessed cardiac arrests, and occurrences of bystander cardiopulmonary resuscitation (CPR). It also encompasses detailed information on ROSC and post-cardiac arrest care, including ECPR, TTM, and percutaneous coronary intervention (PCI). Additionally, it includes data regarding the patients’ final status upon discharge. The authors leveraged these data to investigate the impact of the application of TTM on survival and neurological outcomes in OHCA patients undergoing ECPR. Moreover, to more precisely evaluate the efficacy of TTM and ECPR, the authors restricted their study subjects to refractory cardiac arrest patients who experienced witnessed OHCA of presumed cardiac origin.

This research was meticulously conducted in alignment with the directives enumerated in the Strengthening the Reporting of Observational Studies in Epidemiology checklist, specifically tailored for observational studies. Furthermore, the use of data for this study was authorized by the KCDC in 2021.

### 2.2. Data Source

The OHCAS functions as a registry based on population demographics and a retrospective cohort of patients, which undergoes systematic evaluation by emergency medical services (EMSs). The KCDC provided the clinical data regarding hospital management and outcomes upon discharge of patients with OHCA. The OHCAS registry encompasses extensive data about patients experiencing OHCA, derived from the records of EMSs accessible via the official website of the KCDC. To guarantee both the precision and the completeness of these data, KCDC’s medical record evaluators conducted thorough visits to all emergency departments and healthcare facilities admitting OHCA patients, during which they conducted an in-depth review of patient medical records. The design of the registry’s form was informed by the Utstein-style guidelines and the Resuscitation Outcome Consortium Project, thereby ensuring compliance with recognized standards. 

### 2.3. Study Population

From January 2008 to December 2021, the OHCAS registry recorded a total of 380,239 cases of individuals experiencing OHCA. In this study, we focused on adult patients (aged > 18 years) with witnessed OHCA of presumed cardiac origin, specifically those who did not achieve sustained ROSC (>20 min) in both the pre-hospital and in-hospital stages and were treated with ECPR in the emergency room.

Based on the treatment received, the patients were categorized into two groups: the ECPR without TTM and ECPR with TTM groups. The ECPR with TTM group included patients who were treated with TTM. 

Certain criteria were used to exclude specific subsets of patients with OHCA from the study population. These exclusions included individuals who achieved sustained pre-hospital ROSC (>20 min), had non-cardiac causes of arrest, were <18 years of age, had unwitnessed cardiac arrest, were deceased upon arrival, had a do-not-resuscitate status, had no documented investigation about ECPR or TTM, or had unavailable data on survival or neurological outcomes because of an incomplete medical review or transfer to other healthcare facilities.

### 2.4. Variables

The investigators obtained pertinent information on patient demographics (age and sex), use of bystander CPR, location of cardiac arrest (public versus non-public places), initial cardiac rhythm (shockable, non-shockable, or unknown), time interval from EMS call to ECPR, non-sustained ROSC events, pre-existing comorbidities, and PCI. Comorbidities present before the occurrence of cardiac arrest, such as hypertension, diabetes mellitus, chronic kidney disease, respiratory diseases, and dyslipidemia, were characterized based on clinical diagnoses established by healthcare professionals before the cardiac event and recorded in the patient’s medical histories. A detailed classification of pre-existing comorbidities is provided in Table S1. A shockable rhythm was defined as ventricular fibrillation or pulseless ventricular tachycardia. Cardiac function failure, such as ischemic heart disorders, arrhythmias, cardiac tamponade, or suspected cardiac causes in patients with unexpected arrest, was defined as the cardiac cause of cardiac arrest. A non-sustained ROSC event was defined as any ROSC event before ECPR. 

The assessment of neurological outcomes in this study was conducted using the Glasgow–Pittsburgh Cerebral Performance Category (CPC) scale. Within this framework, a CPC score ranging from 1 to 2 is indicative of good neurological outcomes. Conversely, a score ranging from 3 to 5 on the CPC scale is representative of poor neurological outcomes. A detailed classification of the CPC is provided in Table S2.

The selection and specific approach of TTM were contingent upon the discretion of the attending physicians and the established protocols of their respective hospitals. In the context of TTM, cooling devices were used, defined as any apparatus featuring surface or intravascular cooling capabilities equipped with temperature feedback control mechanisms. Examples of such devices include Arctic Sun^®^ (Medivance Corp, Louisville, KY, USA) and the CoolGard 3000^®^ Thermal Regulation System (Alsius Corporation, Irvine, CA, USA). Furthermore, all TTM protocols implemented within South Korea were in strict compliance with the guidelines set forth by the American Heart Association, specifying a target temperature range of 32–36 °C and a maintenance duration of 12–24 h [14,15] Patients who remained unresponsive following ECPR were identified as potential candidates for TTM. The selection and application of TTM devices were contingent upon the discretion of the physician within the respective hospital. This study’s raw data did not explicitly delineate the duration of the cooling phase or the rate of rewarming used during the TTM procedure. More detailed protocols and information regarding TTM are provided in Table S3.

### 2.5. Outcome Measures

The primary outcome measure in this study was the rate of survival until hospital discharge. Concurrently, the secondary outcome was the incidence of good neurological status, quantified as a CPC score of 1 or 2, assessed at the point of hospital discharge.

### 2.6. Statistical Analysis

In this analysis, descriptive statistical methods were used to encapsulate continuous variables, presenting demographic characteristics in terms of median values and interquartile ranges. Categorical variables were delineated in terms of their frequencies and proportional distributions. The conformity of each continuous variable to a normal distribution was evaluated using the Kolmogorov–Smirnov test. Comparative analysis of categorical variables was conducted using either Pearson’s chi-square test or Fisher’s exact test, as appropriate. For the comparison of continuous variables, the Kruskal–Wallis test was utilized.

To attenuate the influence of potential confounders and selection bias, this study implemented propensity score matching (PSM). This approach was utilized to equilibrate baseline characteristics across the two patient cohorts, thereby enhancing the comparability of the groups. A 1:1 propensity score-matching approach was used with a caliper coefficient of 0.2. Covariates including age, sex, bystander CPR, public places, first documented cardiac rhythm, time interval from EMS call to ECPR, non-sustained ROSC events, pre-existing comorbidities, mechanical CPR, and PCI were included in the matching process. The balance of covariates after PSM was assessed using standardized differences in the means. To appraise the baseline disparity in the covariates of the unmatched and subsequently matched samples, standardized differences were computed. A criterion was set whereby an absolute value lower than 0.1 was indicative of inconsequential variations in either the mean values or prevalence rates of a covariate across the groups under comparison. The matching technique used was that of nearest-neighbor matching, a method that meticulously pairs control and treatment subjects based on the minimal absolute divergence in their estimated propensity scores. Additionally, the influence of targeted temperature management (TTM) on the observed outcomes was rigorously examined via multivariable logistic regression analysis, using a methodical stepwise backward elimination process.

In the context of the multivariable regression analysis, variables that exhibited statistical significance at a threshold of *p* < 0.05 in the univariate analyses were incorporated as covariates. Adjusted odds ratios (ORs) and their corresponding 95% confidence intervals (CIs) were derived utilizing multivariate logistic regression models. The analytic process entailed a stepwise backward elimination methodology within the multivariate logistic regression framework. The entirety of the statistical evaluations was conducted using SPSS software, version 26.0 (IBM, Armonk, NY, USA), in conjunction with the R statistical package, version 3.3.2. The criterion for determining statistical significance was established at a *p*-value of less than 0.05.

The effect of ECPR may vary depending on the timing of ECPR initiation relative to the onset of cardiac arrest. Contemporary guidelines and expert consensus stipulate that the optimal therapeutic window for ECPR intervention falls within a 60-min timeframe following the onset of cardiac arrest [16,17,18,19]. This 60-min threshold is commonly used as a selection criterion in current clinical extracorporeal membrane oxygenation (ECMO) centers. Consequently, we conducted a supplementary subgroup analysis based on the timing of ECPR initiation, categorizing patients into two groups: those receiving ECPR within 1–30 min and those within 31–60 min after cardiac arrest, to assess the impact of TTM by the timing of ECPR. Bystander CPR is widely acknowledged as a significant contributing factor to favorable outcomes in cardiac arrest [20]. Shortening of low-flow duration over time was associated with improved outcomes of ECPR, and the occurrence of non-sustained ROSC may potentially have a positive sign during resuscitation compared with no ROSC [21]. Therefore, we also performed subgroup stratification based on the presence of bystander CPR and the occurrence of non-sustained ROSC to evaluate the influence of TTM on ECPR patients concerning the presence or absence of bystander CPR and the occurrence of non-sustained ROSC.

## 3. Results

### 3.1. Participant Characteristics 

**Figure 1:**
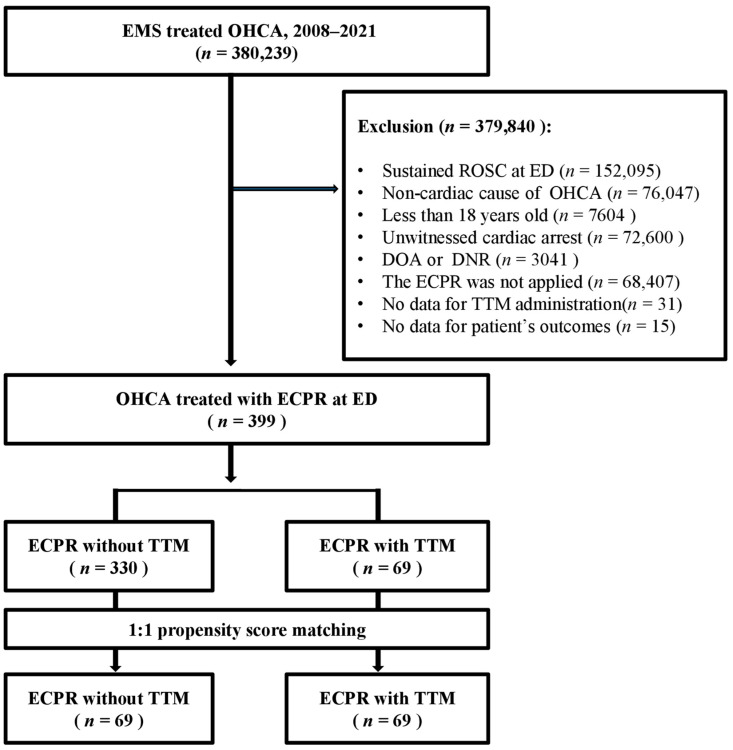
Flow chart of patient inclusions and exclusions in this study. EMS, emergency medical system; OHCA, out-of-hospital cardiac arrest; ROSC, return of spontaneous circulation; ED, emergency department; ECPR, extracorporeal cardiopulmonary resuscitation; TTM, targeted temperature management; DOA, dead on arrival; DNR, do not resuscitate.

During the study period, 399 patients were included in this study (ECPR without TTM, *n* = 330; ECPR with TTM, *n* = 69). Out of 380,239 patients with OHCA, patients with sustained pre-hospital ROSC (*n* = 152,095), non-cardiac cause of OHCA (*n* = 76,047), aged < 18 years (*n* = 7604), unwitnessed cardiac arrest (*n* = 72,600), dead on arrival or do not resuscitate (*n* = 3041), ECPR was not applied (*n* = 68,407), unknown information about TTM (*n* = 31), and unknown survival or neurologic outcomes (*n* = 15) were excluded. The PSM method was used to generate two equivalently matched cohorts, each consisting of 69 patients. These cohorts were differentiated based on the administration of ECPR concomitant with or exclusive of TTM (Figure 1). 

The baseline characteristics of the patients who received ECPR according to TTM administration are presented in Table 1. Patients who received ECPR without TTM were significantly older (58.0 [46.0–66.0] vs. 49.0 [42.0–62.0], *p* = 0.004) and had lower rates of PCI (64.8% vs. 84.1%, *p* = 0.003) than those who received ECPR with TTM (Table 1). Regarding the outcomes at hospital discharge, the ECPR with TTM group had a much higher survival rate than the ECPR without TTM group (26.1% vs. 12.7%, *p* = 0.008) (Table 1).

### 3.2. Propensity Score-Matched Analysis of Outcomes in the Two Groups

Data from the two groups were matched using propensity scores. The comparison of ECPR without TTM vs. ECPR with TTM (*n* = 69 each) is listed in Table 2. The alteration in the absolute standardized difference of the means, coupled with the dot plot depicting the absolute standardized mean difference, demonstrated an enhanced balance of covariates after the implementation of PSM (Figure S1).

### 3.3. Outcome Analysis after PSM

The ECPR with TTM group did not have a higher survival rate (ECPR with TTM, *n* = 16 (23.2%) vs. ECPR without TTM, *n* = 18 (26.1%); *p* = 0.843) or a good neurological outcome (ECPR with TTM, *n* = 10 (14.5%) vs. ECPR without TTM, *n* = 11 (15.9%); *p* = 1.000) compared with the ECPR without TTM group (Table 2). 

### 3.4. Multivariable Logistic Analysis of Outcomes in the Patient Groups after PSM

In terms of survival to hospital discharge, public places and shockable rhythm were significantly associated with improved survival in patients who received ECPR (public places (OR (95% CI), 4.323 (1.541–12.122); *p* = 0.005) and shockable rhythm (OR (95% CI), 10.739 (1.889–61.680); *p* = 0.007). However, no significant difference was observed between ECPR with TTM and ECPR without TTM in these variables (OR (95% CI), 0.930 (0.339–2.546); *p* = 0.887) (Table 3). In neurological outcomes, older age was significantly associated with poor neurological outcomes in patients who received ECPR (OR (95% CI), 0.944 (0.898–0.992); *p* = 0.023). However, no significant differences were observed between ECPR with TTM and ECPR without TTM in this variable (OR (95% CI), 1.139 (0.361–3.593); *p* = 0.824) (Table 3).

### 3.5. Propensity Score-Matched Analysis for Subgroup Analysis of Patient Outcomes According to the Time Interval from EMS Call to the ECPR Pump-on, Bystander CPR, and Non-Sustained ROSC Event

The characteristics of the study population according to TTM administration after PSM in the subgroup analysis are described in Tables S4–S7. The alteration in the absolute standardized difference of the means, coupled with the dot plot depicting the absolute standardized mean difference, demonstrated an enhanced balance in covariates after the implementation of PSM (Figures S1–S4).

### 3.6. Subgroup Analysis for Patient Outcomes by TTM in the Matched Cohort According to the Time Interval from EMS Call to the ECPR Pump-on (EMS Call to ECPR < 30 min or <60 min)

In the subgroup of patients where the time from the EMS call to the initiation of ECPR was < 30 min, no statistically significant difference was observed in survival to hospital discharge between the ECPR with TTM and ECPR without TTM groups (17.1% vs. 28.6%; *p* = 0.393). Similarly, no significant difference was observed in the percentage of good neurological outcomes between the two groups (11.4% vs. 17.1%; *p* = 0.733) (Table 4 and Table S4).

In the subgroup of patients where the time from the EMS call to the initiation of ECPR was <60 min, no statistically significant difference was observed in survival to hospital discharge between the ECPR with TTM group and the ECPR without TTM group (32.6% vs. 26.1%; *p* = 0.471). Similarly, no significant difference was observed in the percentage of good neurological outcomes between the two groups (19.6% vs. 15.2%, *p* = 0.104) (Table 4 and Table S5).

### 3.7. Subgroup Analysis for Patient Outcomes by TTM in the Matched Cohort According to Bystander CPR or Non-Sustained ROSC Event

Table 5 presents the patient outcomes in the matched cohort, stratified by whether the patients received bystander CPR or experienced a non-sustained ROSC event and whether they were treated with ECPR with or without TTM. In this matched cohort, the addition of TTM to ECPR did not significantly affect survival or neurological outcomes for patients with OHCA, regardless of whether they received bystander CPR (survival: 17.1% vs. 28.6%, *p* = 0.393) (neurological outcome: 11.4% vs. 17.1%, *p* = 0.733) or experienced a non-sustained ROSC event (survival 19.6% vs. 15.2%, *p* = 0.104) (neurological outcome: 19.6% vs. 15.2%, *p* = 0.104).

## 4. Discussion

The current study found that the propensity score analysis of all eligible patients showed no association between TTM and improved outcomes in patients who underwent ECPR for OHCA.

Among patients with OHCA, ECPR has emerged as a potential life-saving strategy [22]. TTM denotes the deliberate reduction in a patient’s body temperature to alleviate brain damage following cardiac arrest [9]. Although both therapies have demonstrated potential individually, the combined impact of ECPR and TTM on clinical outcomes is currently being actively researched [12]. This study aimed to evaluate whether incorporating TTM into ECPR improves clinical outcomes in patients with OHCA. This study’s results revealed no substantial enhancement in survival or neurological outcomes between ECPR with and without TTM.

In recent studies on the application of TTM in patients who received ECPR [10,11,12,13], Huang et al. reported that patients with refractory cardiac arrest who underwent ECPR with TTM did not have better outcomes than those who underwent ECPR without TTM; however, the results were limited by the heterogeneity in the included studies in the systematic review and meta-analysis [11]. Sakurai et al. reported the potential superiority of ECPR with TTM over ECPR without TTM in adult patients with OHCA [12]. However, it is noteworthy that their study included patients with witnessed OHCA undergoing ECPR, irrespective of the origin of the arrest. In contrast, our study specifically enrolled patients with witnessed OHCA of presumed cardiac origin who were subsequently subjected to ECPR. This divergence in study populations may have contributed to the observed disparity in the impact of TTM on patients who received ECPR between the two studies.

The lack of significant enhancements in the outcomes of patients who received ECPR treated with TTM in this study may be related to the condition of refractory cardiac arrest and the low rate of bystander CPR. Although modern resuscitation techniques, such as ECMO, are used for patients with refractory cardiac arrest, severe brain damage may have already occurred before ECPR was applied [23,24]. The low rate of bystander CPR observed among patients undergoing ECPR, despite the context of witnessed OHCA, is notably suboptimal, denoting an escalation in the no-flow time [24,25,26]. This increased duration of the low-flow state emerges as an adverse determinant of patient outcomes [24]. Consequently, a nuanced subgroup analysis was performed to isolate patients who received bystander CPR selectively. Nevertheless, even in this subset of patients benefiting from bystander CPR, the implementation of TTM failed to exert a discernible influence on ECPR recipient outcomes.

Moreover, the use of ECPR in these individuals may include elevated susceptibility to consequences such as hemorrhage, infection, and blood clot formation [27]. Although complications of ECPR were not reported in the raw data of the study, we hypothesized that the implementation of TTM could potentially increase bleeding complications, such as intracranial hemorrhage, due to the reduction in body temperature [28,29]. This assumption is based on the fact that the use of anticoagulation with an ECMO circuit may increase bleeding risk [30,31]. 

Another potential determinant is the operational mechanism of TTM devices utilized in patients undergoing ECPR. Given that venoarterial ECMO operates by removing blood from the patient’s body, running it through a machine to oxygenate it, removing carbon dioxide, and then returning it to the patient [32], using ECMO in this manner may similarly impact TTM devices. TTM utilizes two primary categories of devices: endovascular cooling devices and surface cooling devices equipped with temperature feedback control mechanisms [33]. Endovascular cooling devices utilize convection as a cooling mechanism to enable efficient heat transfer between a catheter inserted into the vena cava and the patient’s bloodstream [34]. In contrast, surface cooling devices facilitate the passage of heat among tissues, effectively transferring heat from the body’s central region to the outer surface [34]. Given these methods, TTM may demonstrate less efficacy when ECMO is utilized because ECMO entails the extraction of blood from the patient’s body via a major vein, followed by its reinfusion. Therefore, we propose that the neuroprotective benefits of TTM may be diminished in patients receiving ECPR compared with post-cardiac arrest patients with spontaneous circulation. In addition, ECMO machines can integrate heat exchangers to decrease blood temperature to a specified desired range, usually 32–36 °C, without TTM devices [35,36,37,38,39]. In other words, ECMO may have similar effects to TTM, even without using TTM. Therefore, using TTM devices such as surface cooling devices or endovascular cooling devices may result in only a slight improvement in patient outcomes.

The effect of ECPR may vary depending on when it is initiated at the onset of cardiac arrest [16,17,18,19]. In this study, the median time to ECPR initiation was 30 min. Two subgroup analyses, categorized by the timing of ECPR initiation (<30 min and 31–60 min), revealed that TTM had no impact on the clinical outcomes of patients who received ECPR. Based on the guidelines that state that ECPR should be initiated within 60 min of the patient’s collapse [16,17,18,19], this study found no significant difference in patient outcomes between the two groups with relatively shorter periods of reduced blood flow to the heart (<30 min and 31–60 min). These findings suggest that the benefits of using TTM in patients receiving ECPR may be minimal, regardless of the duration of reduced blood flow within 60 min of ECPR initiation from the patient’s collapse.

Furthermore, certain characteristics, such as the mental condition and level of severity of patients who underwent ECPR before TTM, were not evaluated due to limitations in the available data. Nevertheless, it is wise to recognize the possible influence of these unanalyzed variables when interpreting the results documented in this study. Based on the guidelines that state that ECPR should be initiated within 60 min of the patient’s collapse [16,17,18,19], the outcomes may have been influenced by variability in TTM onset and duration, as it is possible that earlier or longer TTM may be necessary to obtain maximal benefit [40]. Hence, it is imperative to thoroughly assess the potential hazards and advantages linked to TTM and ECPR on a case-by-case basis.

This study has several limitations. First, although the fact that this study included a nationwide cohort, this study’s retrospective approach may have produced some bias. Second, survival and neurological outcomes were assessed upon discharge from the hospital. It is important to recognize that the results may continue to develop beyond this specified period, perhaps altering after 6 months or 1 year. Third, despite the lack of evidence from the propensity score analysis regarding the effectiveness of TTM, it is crucial to acknowledge that additional uncontrolled variables could influence outcomes and introduce bias. Fourth, 38.6% of the patients exhibited an “unknown” initial cardiac rhythm at the EMS stage, signifying the absence of precise electrocardiogram assessments during this phase. This introduces an element of uncertainty regarding whether the initial cardiac rhythm might influence the outcomes of patients who undergo TTM. Finally, our data did not demonstrate the efficacy of TTM in patients who underwent ECPR using ECPR and TTM methods from different countries.

## 5. Conclusions

TTM may not be advisable for improving outcomes in ECPR patients with witnessed OHCA of presumed cardiac origin when compared with ECPR without TTM. Randomized controlled studies are necessary to assess these findings, incorporating more detailed TTM information such as the targeted temperature. 

## Figures and Tables

**Table 1:** Characteristics of the study population according to the administration of TTM.

Variables	Total	ECPR without TTM	ECPR with TTM	*p*-Value
	(*n* = 399)	(*n* = 330)	(*n* = 69)	
**Age, years [median (IQR)]**	56.0 [45.0–65.0]	58.0 [46.0–66.0]	49.0 [42.0–62.0]	0.004
**Sex, male, n (%)**	331 (83.0%)	271 (82.1%)	60 (87.0%)	0.426
**Bystander CPR**	161 (40.4%)	130 (39.4%)	31 (44.9%)	0.473
**Location of cardiac arrest**				
Public places	100 (25.1%)	86 (26.1%)	14 (20.3%)	0.394
**First cardiac rhythm at EMS**	91 (22.8%)	80 (24.2%)	11 (15.9%)	0.292
Shockable rhythms, n (%)	114 (28.6%)	91 (27.6%)	23 (33.3%)	
Non-shockable rhythms, n (%)	91 (22.8%)	80 (24.2%)	11 (15.9%)	
Unknown	194 (48.6%)	159 (48.2%)	35 (50.7%)	
EMS call to ECPR, mins *	37.0 [20.2–77.0]	42.5 [20.1–80.0]	30.0 [20.2–66.0]	0.277
**Non-sustained ROSC event ^a^**	229 (57.4%)	186 (56.4%)	43 (62.3%)	0.438
**Pre-existing comorbidity, n (%)**				
HTN	157 (39.3%)	133 (40.3%)	24 (34.8%)	0.473
DM	102 (25.6%)	88 (26.7%)	14 (20.3%)	0.341
Heart disease	85 (21.3%)	74 (22.4%)	11 (15.9%)	0.301
Chronic kidney disease	11 (2.8%)	10 (3.0%)	1 (1.4%)	0.698
Respiratory disease	10 (2.5%)	10 (3.0%)	0 (0.0%)	0.222
Stroke	15 (3.8%)	14 (4.2%)	1 (1.4%)	0.485
Dyslipidemia	33 (8.3%)	28 (8.5%)	5 (7.2%)	0.921
**Mechanical CPR**	119 (29.8%)	102 (30.9%)	17 (24.6%)	0.373
**Post-cardiac arrest care**				
PCI	272 (68.2%)	214 (64.8%)	58 (84.1%)	0.003
**Outcomes at hospital discharge**				
Survival	60 (15.0%)	42 (12.7%)	18 (26.1%)	0.008
Good neurological outcome	39 (9.8%)	28 (8.5%)	11 (15.9%)	0.094

Abbreviations: TTM, targeted temperature management; IQR, interquartile range; CPR, cardiopulmonary resuscitation; EMS, emergency medical service; ECPR, extracorporeal cardiopulmonary resuscitation; ROSC, return of spontaneous circulation; DM, diabetes mellitus; HTN, hypertension; PCI, percutaneous coronary intervention. * Time from EMS call to EMMO pump-on at ED. ^a^ Any ROSC event before ECPR.

**Table 2:** Characteristics of the study population according to administration of TTM after propensity score.

Variables	Total	ECPR without TTM	ECPR with TTM	*p*-Value
	(*n* = 138)	(*n* = 69)	(*n* = 69)	
**Age, years [median (IQR)]**	50.0 [41.0–60.0]	50.0 [41.0–58.0]	49.0 [42.0–62.0]	0.606
**Sex, male, n (%)**	117 (84.8%)	57 (82.6%)	60 (87.0%)	0.636
**Bystander CPR**	63 (45.7%)	32 (46.4%)	31 (44.9%)	1.000
**Location of cardiac arrest**				
Public places	33 (23.9%)	19 (27.5%)	14 (20.3%)	0.425
**First cardiac rhythm at EMS**	27 (19.6%)	16 (23.2%)	11 (15.9%)	0.055
Shockable rhythms, n (%)	34 (24.6%)	11 (15.9%)	23 (33.3%)	
Non-shockable rhythms, n (%)	27 (19.6%)	16 (23.2%)	11 (15.9%)	
Unknown	77 (55.8%)	42 (60.9%)	35 (50.7%)	
EMS call to ECPR, mins *	30.0 [10.9–62.0]	30.6 [10.8–57.0]	30.0 [20.2–66.0]	0.069
**Non-sustained ROSC event ^a^**	87 (63.0%)	44 (63.8%)	43 (62.3%)	1.000
**Pre-existing comorbidity, n (%)**				
HTN	43 (31.2%)	19 (27.5%)	24 (34.8%)	0.462
DM	30 (21.7%)	16 (23.2%)	14 (20.3%)	0.836
Heart disease	27 (19.6%)	16 (23.2%)	11 (15.9%)	0.391
Chronic kidney disease	3 (2.2%)	2 (2.9%)	1 (1.4%)	1.000
Respiratory disease	0 (0.0%)	0 (0.0%)	0 (0.0%)	1.000
Stroke	3 (2.2%)	2 (2.9%)	1 (1.4%)	1.000
Dyslipidemia	10 (7.2%)	5 (7.2%)	5 (7.2%)	1.000
**Mechanical CPR**	30 (21.7%)	13 (18.8%)	17 (24.6%)	0.536
**Post-cardiac arrest care**				
PCI	113 (81.9%)	55 (79.7%)	58 (84.1%)	0.658
**Outcomes at hospital discharge**				
Survival	34 (24.6%)	16 (23.2%)	18 (26.1%)	0.843
Good neurological outcome	21 (15.2%)	10 (14.5%)	11 (15.9%)	1.000

Abbreviations: TTM, targeted temperature management; IQR, interquartile range; CPR, cardiopulmonary resuscitation; EMS, emergency medical service; ECPR, extracorporeal cardiopulmonary resuscitation; ROSC, return of spontaneous circulation; DM, diabetes mellitus; HTN, hypertension; PCI, percutaneous coronary intervention. * Time from EMS call to EMMO pump-on at ED. ^a^ Any ROSC event before ECPR.

**Table 3:** Multivariable logistic regression analysis for outcomes in propensity-matched patients.

	OR (95%CI)	*p*-Value
Survival to hospital discharge *		
Public places	4.323 (1.541–12.122)	0.005
Shockable rhythm	10.739 (1.889–61.680)	0.007
TTM	0.930 (0.339–2.546)	0.887
Good neurologic outcome *		
Age	0.944 (0.898–0.992)	0.023
TTM	1.139 (0.361–3.593)	0.824

The model of the multivariate logistic regression analysis is stepwise backward elimination. ROSC, return of spontaneous circulation; OR, adjusted odds ratio; CI, confidence interval. * Adjusted odds ratio for age, sex, bystander CPR, public places, initial documented cardiac rhythm, total CPR duration, any ROSC event, diabetes mellitus, hypertension, heart disease, chronic kidney disease, respiratory disease, mechanical CPR, and percutaneous coronary intervention.

**Table 4:** Outcomes of patients by TTM in the matched cohort according to the time interval from EMS call to ECPR after PSM.

	EMS Call to ECPR Pump-on < 30 min			EMS Call to ECPR Pump-on < 60 min		
	ECPR without TTM, n/N (%)	ECPR with TTM, n/N (%)	*p*	ECPR without TTM, n/N (%)	ECPR with TTM, n/N (%)	*p*
		
**Survival to hospital discharge**	6/35 (17.1%)	10/35 (28.6%)	0.393	15/46 (32.6%)	12/46 (26.1%)	0.471
**Good neurological outcome**	4/35 (11.4%)	6/35 (17.1%)	0.733	9/46 (19.6%)	7/46 (15.2%)	0.104

Abbreviations: TTM, targeted temperature management; EMS, emergency medical service; ECPR, extracorporeal cardiopulmonary resuscitation; PSM, propensity score matching.

**Table 5:** Outcomes of patients by TTM in the matched cohort according to bystander CPR or non-sustained ROSC event after PSM.

	Bystander CPR			Non-Sustained ROSC Event ^a^		
	ECPR without TTM, n/N (%)	ECPR with TTM, n/N (%)	*p*	ECPR without TTM, n/N (%)	ECPR with TTM, n/N (%)	*p*
		
**Survival to hospital discharge**	7/30 (23.3%)	9/30 (30.0%)	0.770	14/43 (32.6%)	10/43 (23.3%)	0.471
**Good neurological outcome**	6/30 (20.0%)	3/30 (10.0%)	0.472	12/43 (27.9%)	5/43 (11.6%)	0.104

Abbreviations: TTM, targeted temperature management; ECPR, extracorporeal cardiopulmonary resuscitation; ROSC, return of spontaneous circulation; PSM, propensity score matching. ^a^ Any ROSC event before ECPR.

## Data Availability

The authors utilized the database made available by the Korea Disease Control and Prevention Agency, which holds the authority over the OHCA registry dataset in Korea. Access to this dataset requires permission, and interested parties can request access through the official website (https://www.kdca.go.kr/injury/biz/injury/main/mainPage.do, accessed on 20 September 2023).

## Supplementary Materials

The following supporting information can be downloaded at: https://www.mdpi.com/article/10.3390/jpm14020185/s1, Figure S1: Alteration in the absolute standardized difference in means (A) and a dot plot representation of the absolute standardized mean difference (B) among patients receiving ECPR without TTM, and those undergoing ECPR without TTM, both prior to and subsequent to the application of propensity score matching; Figure S2: Alteration in the absolute standardized difference in means (A) and a dot plot representation of the absolute standardized mean difference (B) among patients receiving ECPR without TTM, and those undergoing ECPR without TTM, both prior to and subsequent to the application of propensity score matching in subgroups where the time interval from the EMS call to ECPR initiation was less than 30 min; Figure S3: Alteration in the absolute standardized difference in means (A) and a dot plot representation of the absolute standardized mean difference (B) among patients receiving ECPR without TTM, and those undergoing ECPR without TTM, both prior to and subsequent to the application of propensity score matching in the subgroup where the time interval from the EMS call to ECPR initiation was less than 60 min; Figure S4: Alteration in the absolute standardized difference in means (A) and a dot plot representation of the absolute standardized mean difference (B) among patients receiving ECPR without TTM, and those undergoing ECPR without TTM, both prior to and subsequent to the application of propensity score matching in the subgroup that had bystander CPR; Figure S5: Alteration in the absolute standardized difference in means (A) and a dot plot representation of the absolute standardized mean difference (B) among patients receiving ECPR without TTM, and those undergoing ECPR without TTM, both prior to and subsequent to the application of propensity score matching in subgroups that had a non-sustained ROSC event; Table S1: Definition and detailed classification of pre-existing comorbidity; Table S2: Glasgow–Pittsburgh Cerebral Performance Categories (CPC) scores and their corresponding descriptions; Table S3: Protocol and information about targeted temperature management (TTM); Table S4: Characteristics of the study population according to administration of TTM after propensity score matching in the subgroup where the time interval from the EMS call to ECPR initiation was less than 30 min; Table S5: Characteristics of the study population according to the administration of TTM after propensity score matching in the subgroup where the time interval from the EMS call to ECPR initiation was less than 60 min; Table S6: Characteristics of the study population according to administration of TTM after propensity score matching in the subgroup that had bystander CPR; Table S7: Characteristics of the study population according to administration of TTM after propensity score matching in the subgroup that had a non-sustained ROSC event prior to ECPR.
