# Supplementary material for: Target Temperature Management Effect on the Clinical Outcome of Patients with Out-of-Hospital Cardiac Arrest Treated with Extracorporeal Cardiopulmonary Resuscitation: A Nationwide Observational Study
Source: J Pers Med. 2024 Feb 7;14(2):185. doi: 10.3390/jpm14020185 (PMC10890305; doi:10.3390/jpm14020185)
Supplement: Supplementary file 1 [file jpm-14-00185-s001.zip › jpm-2838426-supplementary.pdf]

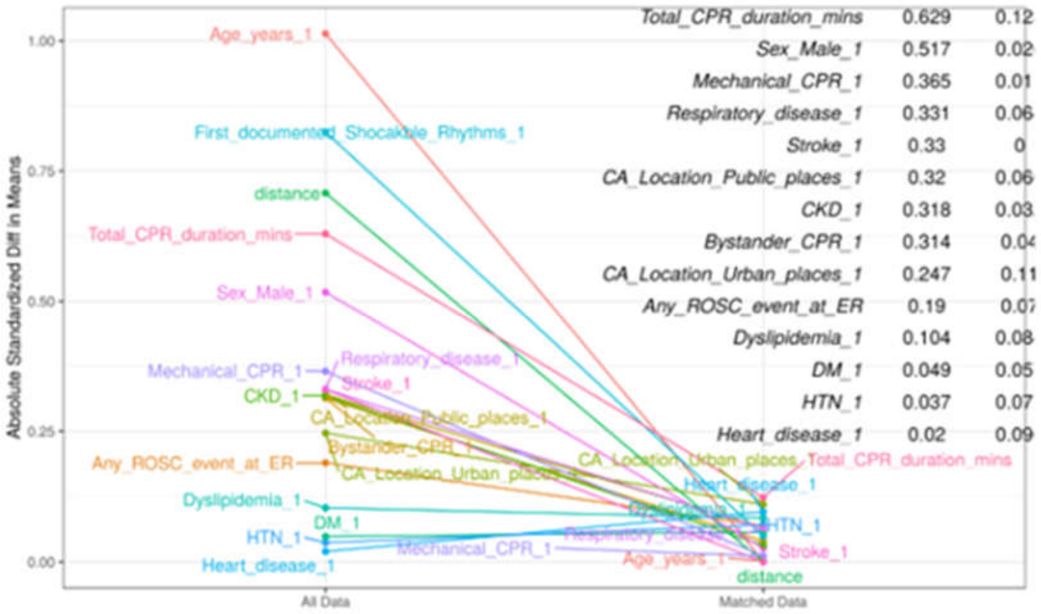

(A)

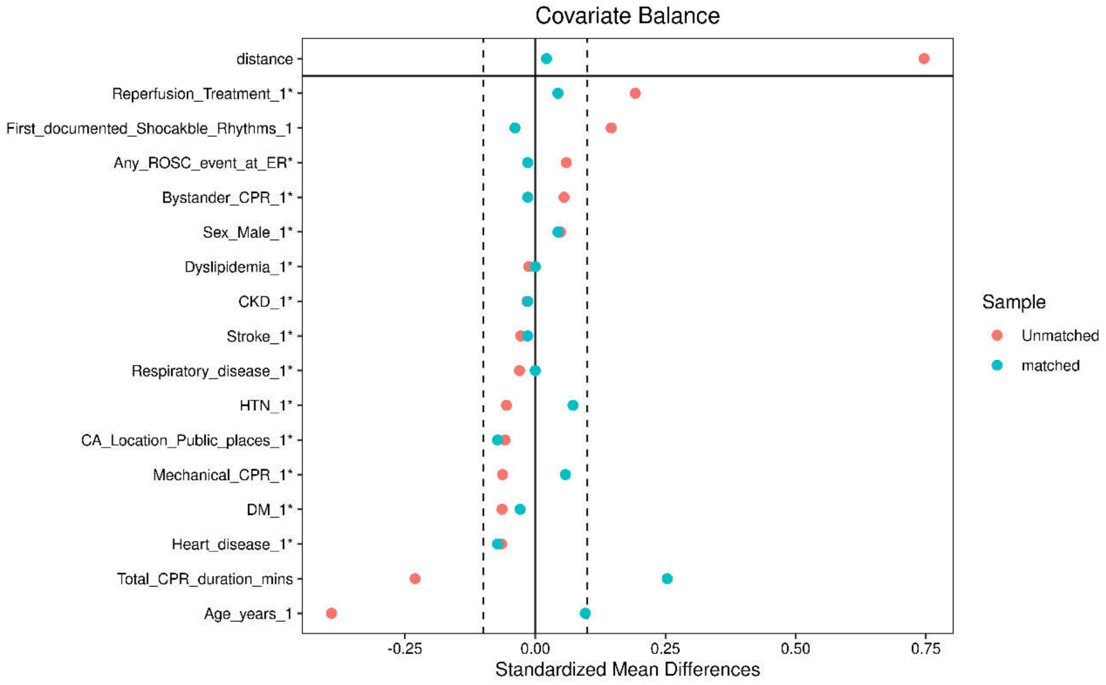

(B)

Figure S1. Alteration in the Absolute Standardized Difference in Means (A) and a Dot Plot Representation of the Absolute Standardized Mean Difference (B) among patients receiving ECPR without TTM, and those undergoing ECPR without TTM, both prior to and subsequent to the application of propensity score matching.

Abbreviations: CA, cardiac arrest; CPR, cardiopulmonary resuscitation; CKD, chronic kidney disease; DM, diabetes mellitus; HTN, hypertension; ROSC, return of spontaneous circulation; ER, emergency room, TTM, targeted temperature management; ECPR, extracorporeal cardiopulmonary resuscitation.

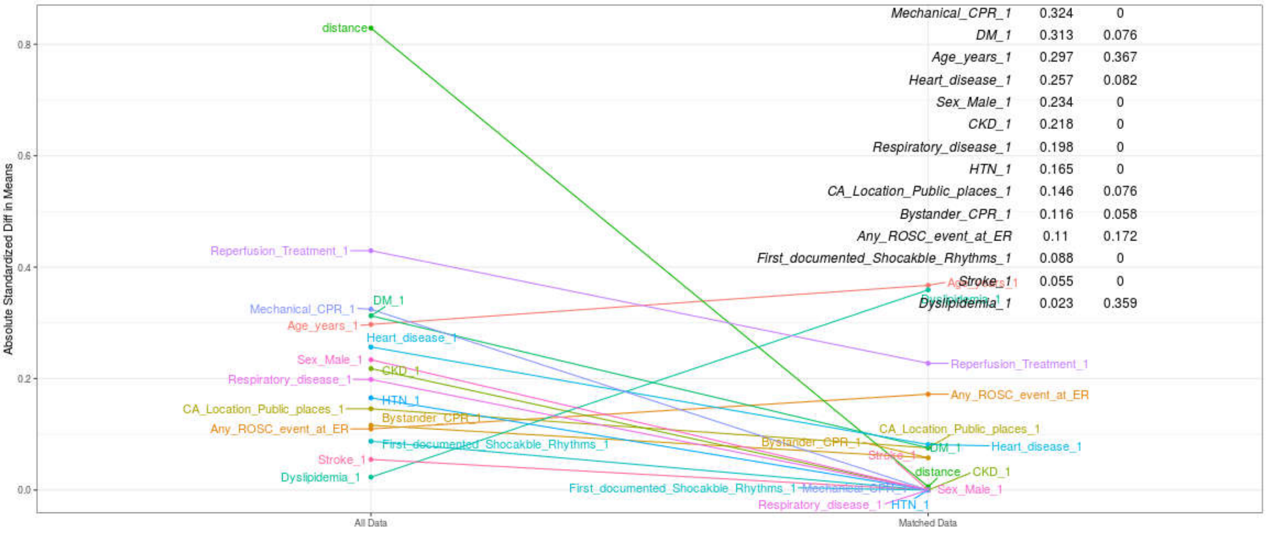

(A)

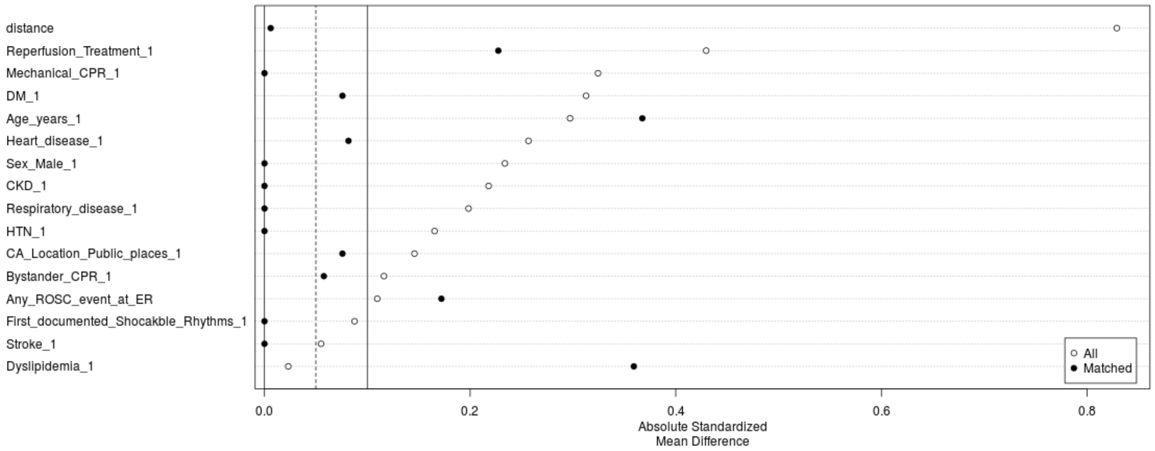

(B)

Figure S2. Alteration in the Absolute Standardized Difference in Means (A) and a Dot Plot Representation of the Absolute Standardized Mean Difference (B) among patients receiving ECPR without TTM, and those undergoing ECPR without TTM, both prior to and subsequent to the application of propensity score matching in subgroup where the time interval from the EMS call to ECPR initiation was less than 30 minutes.

Abbreviations: CA, cardiac arrest; CPR, cardiopulmonary resuscitation; CKD, chronic kidney disease; DM, diabetes mellitus; HTN, hypertension; ROSC, return of spontaneous circulation; ER, emergency room, TTM, targeted temperature management; ECPR, extracorporeal cardiopulmonary resuscitation; EMS, emergency medical services.

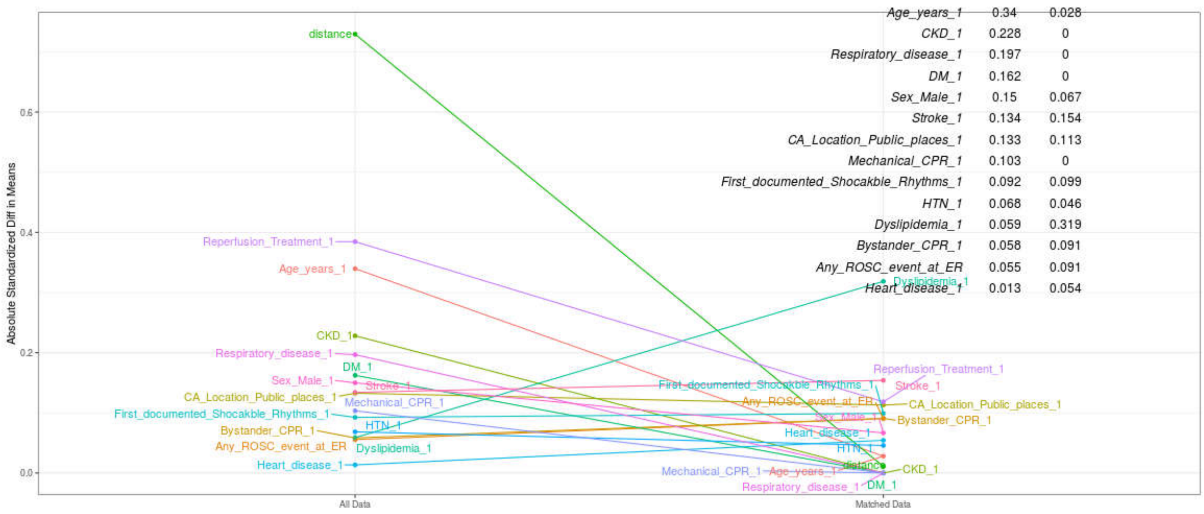

(A)

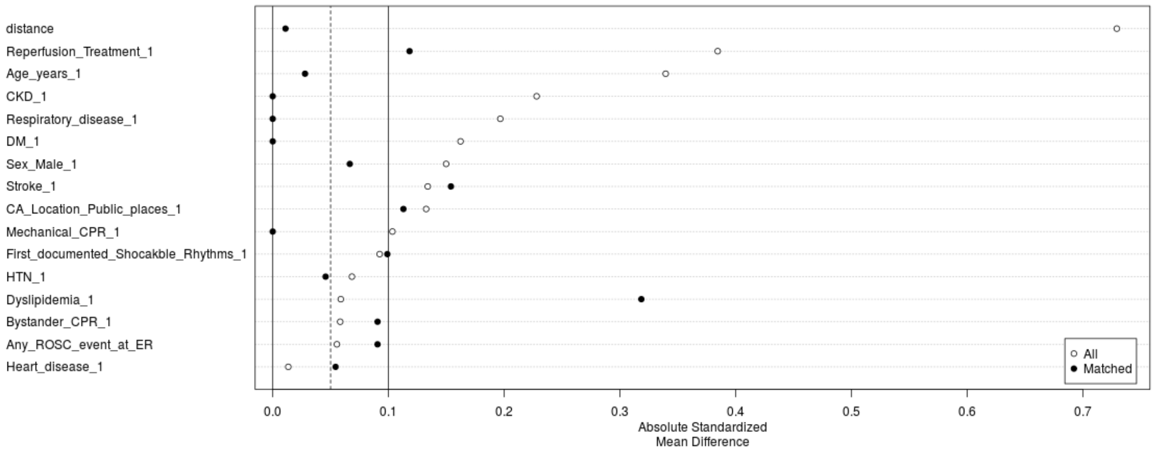

(B)

Figure S3. Alteration in the Absolute Standardized Difference in Means (A) and a Dot Plot Representation of the Absolute Standardized Mean Difference (B) among patients receiving ECPR without TTM, and those undergoing ECPR without TTM, both prior to and subsequent to the application of propensity score matching in subgroup where the time interval from the EMS call to ECPR initiation was less than 60 minutes.

Abbreviations: CA, cardiac arrest; CPR, cardiopulmonary resuscitation; CKD, chronic kidney disease; DM, diabetes mellitus; HTN, hypertension; ROSC, return of spontaneous circulation; ER, emergency room, TTM, targeted temperature management; ECPR, extracorporeal cardiopulmonary resuscitation; EMS, emergency medical services.

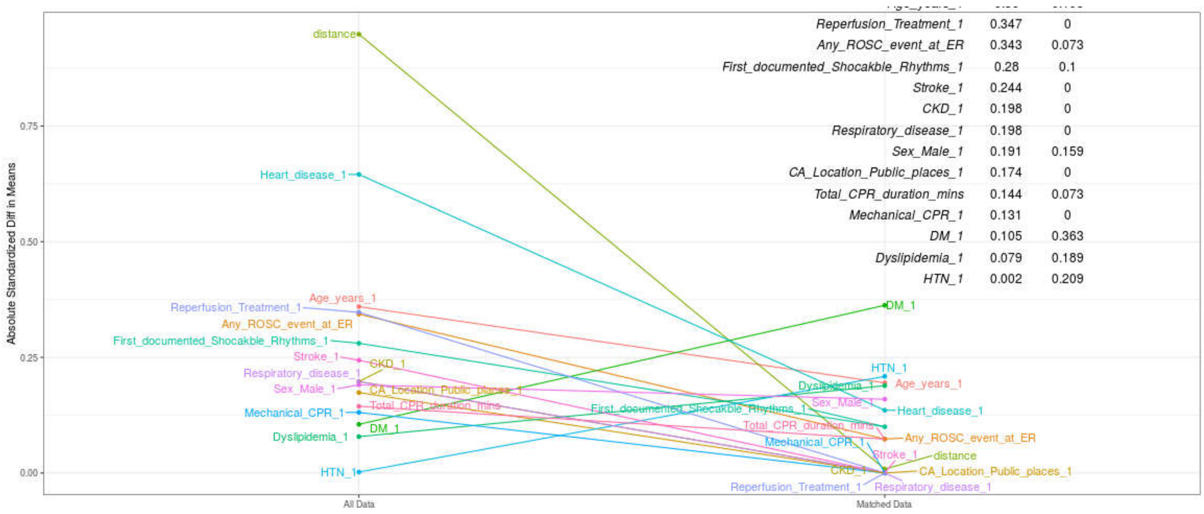

(A)

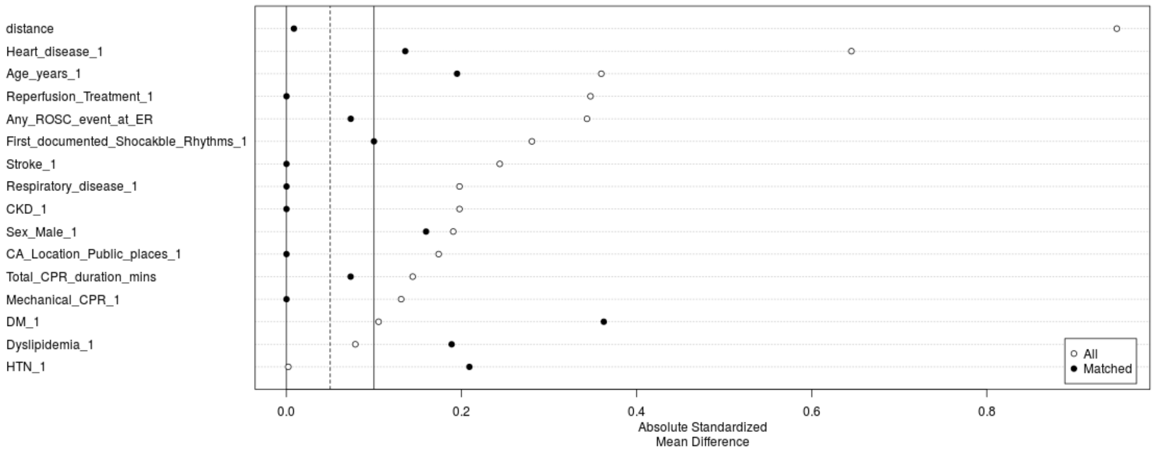

(B)

Figure S4. Alteration in the Absolute Standardized Difference in Means (A) and a Dot Plot Representation of the Absolute Standardized Mean Difference (B) among patients receiving ECPR without TTM, and those undergoing ECPR without TTM, both prior to and subsequent to the application of propensity score matching in subgroup had bystander CPR.

Abbreviations: CA, cardiac arrest; CPR, cardiopulmonary resuscitation; CKD, chronic kidney disease; DM, diabetes mellitus; HTN, hypertension; ROSC, return of spontaneous circulation; ER, emergency room, TTM, targeted temperature management; ECPR, Extracorporeal cardiopulmonary resuscitation.

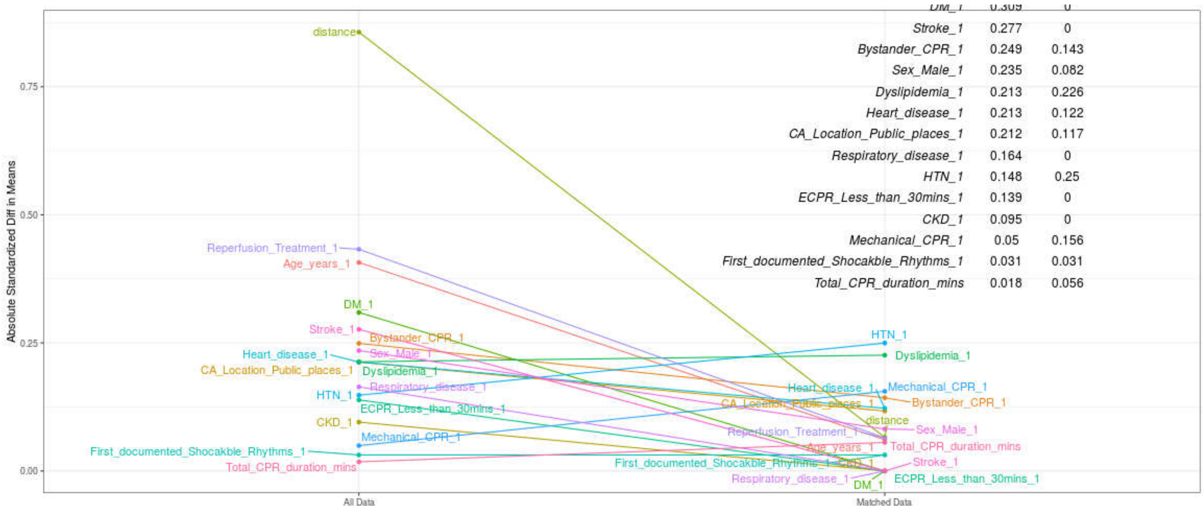

(A)

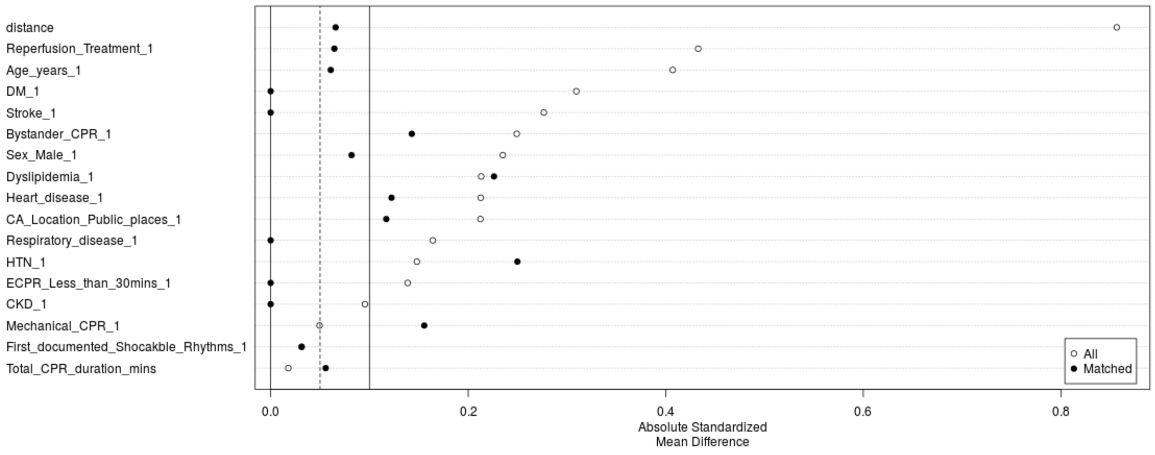

(b)

Figure S5. Alteration in the Absolute Standardized Difference in Means (A) and a Dot Plot Representation of the Absolute Standardized Mean Difference (B) among patients receiving ECPR without TTM, and those undergoing ECPR without TTM, both prior to and subsequent to the application of propensity score matching in subgroup had non-sustained ROSC event.

Abbreviations: CA, cardiac arrest; CPR, cardiopulmonary resuscitation; CKD, chronic kidney disease; DM, diabetes mellitus; HTN, hypertension; ROSC, return of spontaneous circulation; ER, emergency room, TTM, targeted temperature management; ECPR, Extracorporeal cardiopulmonary resuscitation.

Table S1. Definition and detailed classification of pre-existing comorbidity

Disease	Definition and detailed classification
Hypertension	The disease was diagnosed by the doctor prior to cardiac arrest and is clearly stated in the medical record
Diabetes mellitus	The disease was diagnosed by the doctor prior to cardiac arrest and is clearly stated in the medical record.
Heart disease	The disease was diagnosed by the doctor prior to cardiac arrest and is clearly stated in the medical record. (Ischemic heart disease, myocardial infarction, angina, valvular heart disease, arrhythmia, congestive heart disease, myocardiopathy, congenital heart disease, previous heart intervention record including stent, primary coronary intervention, coronary artery bypass grafting, and other heart disease)
Chronic renal disease	The disease was diagnosed by the doctor prior to cardiac arrest and is clearly stated in the medical record. (chronic renal failure, dialysis, kidney transplantation, and other chronic kidney disease)
Chronic respiratory disease	The disease was diagnosed by the doctor prior to cardiac arrest and is clearly stated in the medical record. (Asthma, chronic obstructive pulmonary disease, pulmonary tuberculosis, other chronic respiratory disease.)
Stroke	The disease was diagnosed by the doctor prior to cardiac arrest and is clearly stated in the medical record. (cerebral infarction, cerebral haemorrhage, and unclassified stroke)
Dyslipidemia	The disease was diagnosed by the doctor prior to cardiac arrest and is clearly stated in the medical record. (hyperlipidaemia and unclassified dyslipidemia)

Table S2. Glasgow-Pittsburgh Cerebral Performance Categories (CPC) scores and their corresponding descriptions

CPC Score	Description
CPC 1	Good cerebral performance with mild or no neurological deficit
CPC 2	Moderate cerebral disability with moderate neurological deficit, sufficient functional independence for activities of daily living
CPC 3	Severe cerebral disability with severe neurological deficit, dependence on others for daily activities
CPC 4	Coma or vegetative state, absence of meaningful neurological function, inability to interact with the environment
CPC 5	Brain death or death due to neurological injury

Table S3. Protocol and information about targeted temperature management

All patients who experienced out-of-hospital and remained unresponsive after ECPR were identified as potential candidates for targeted temperature management (TTM). However, the duration of cooling and the rate of rewarming during the TTM process were not explicitly reported from the raw data of this study.

The application of TTM protocols relied on the discretion of the attending physicians, with the choice of external cooling devices (Artic Sun, Gaymar III, Blanketrol III) or internal cooling devices (Coolgard). The specific target temperature for each patient was determined in accordance with the TTM protocol established at the participating hospital.

In the absence of specific contraindications or issues, this study primarily maintained a state of moderate hypothermia within the range of 32–34°C. In cases where patients exhibited hemodynamic instability a shift towards mild hypothermia within the range of 35–36°C was generally implemented.

The distribution of targeted body temperatures and the corresponding number of patients in this cohort were as follows:

**Targeted body temperature**	**Number of patients**
33.0	23
33.5	2
34.0	20
34.5	1
35.0	6
35.5	1
36.0	12
36.5	1
unknown	3
**Total**	**69**

**Type of cooling devices**	**Number of patients**
External cooling devices	42
Internal cooling devices	4
Unknown specific type of cooling devices	23
**Total**	**69**

Table S4. Characteristics of the study population according to administration of TTM after propensity score matching in subgroup where the time interval from the EMS call to ECPR initiation was less than 30 minutes.

Variables	Total<br>(N=70)	ECPR without TTM<br>(N=35)	ECPR with TTM<br>(N=35)	p
**Age, years [median (IQR)]**	53.0 [45.0;62.0]	57.0 [48.0;63.0]	50.0 [42.0;61.5]	0.152
**Sex, male, n (%)**	62 (88.6%)	31 (88.6%)	31 (88.6%)	1.000
**Bystander CPR**	29 (41.4%)	14 (40.0%)	15 (42.9%)	1.000
**Location of cardiac arrest**				
Public places	13 (18.6%)	7 (20.0%)	6 (17.1%)	1.000
**First cardiac rhythm at EMS**				1.000
Shockable rhythms, n (%)	0 (0.0%)	0 (0.0%)	0 (0.0%)	
Non-shockable rhythms, n (%)	0 (0.0%)	0 (0.0%)	0 (0.0%)	
Unknown	70 (100.0%)	35 (100.0%)	35 (100.0%)	
**Non-sustained ROSC event**[a]	41 (58.6%)	22 (62.9%)	19 (54.3%)	0.627
**Pre-existing comorbidity, n (%)**				
HTN	24 (34.3%)	12 (34.3%)	12 (34.3%)	1.000
DM	13 (18.6%)	7 (20.0%)	6 (17.1%)	1.000
Heart disease	11 (15.7%)	6 (17.1%)	5 (14.3%)	1.000
Chronic kidney disease	0 (0.0%)	0 (0.0%)	0 (0.0%)	1.000
Respiratory disease	0 (0.0%)	0 (0.0%)	0 (0.0%)	1.000
Stroke	2 (2.9%)	1 (2.9%)	1 (2.9%)	1.000
Dyslipidemia	12 (17.1%)	8 (22.9%)	4 (11.4%)	0.341
**Mechanical CPR**	16 (22.9%)	8 (22.9%)	8 (22.9%)	1.000
**Post-cardiac arrest care**				
PCI	61 (87.1%)	32 (91.4%)	29 (82.9%)	0.477
**Outcomes at hospital discharge**				
Survival	16 (22.9%)	6 (17.1%)	10 (28.6%)	0.393
Good neurological outcome	10 (14.3%)	4 (11.4%)	6 (17.1%)	0.733

Abbreviations: TTM, targeted temperature management; IQR, interquartile range; CPR, cardiopulmonary resuscitation; EMS, emergency medical service; ECPR, extracorporeal cardiopulmonary resuscitation; ROSC, return of spontaneous circulation; ER; emergency room; DM, diabetes mellitus; HTN, hypertension; PCI, percutaneous coronary intervention..

[a] Any non-sustained ROSC event prior to ECPR

Table S5. Characteristics of the study population according to administration of TTM after propensity score matching in subgroup where the time interval from the EMS call to ECPR initiation was less than 60 minutes.

**Variables**	**Total (N=92)**	**ECPR without TTM (N=46)**	**ECPR with TTM (N=46)**	p
**Age, years [median (IQR)]**	50.0 [39.5-62.0]	50.0 [38.0-60.0]	50.5 [40.0-63.0]	0.489
**Sex, male, n (%)**	80 (87.0%)	40 (87.0%)	40 (87.0%)	1.000
**Bystander CPR**	33 (35.9%)	14 (30.4%)	19 (41.3%)	0.385
**Location of cardiac arrest**				1.000
Public places	17 (18.5%)	8 (17.4%)	9 (19.6%)	
**First cardiac rhythm at EMS**				0.693
Shockable rhythms, n (%)	12 (13.0%)	7 (15.2%)	5 (10.9%)	
Non-shockable rhythms, n (%)	10 (10.9%)	4 (8.7%)	6 (13.0%)	
Unknown	70 (76.1%)	35 (76.1%)	35 (76.1%)	
**Non-sustained ROSC event** [a]	50 (54.3%)	23 (50.0%)	27 (58.7%)	0.530
**Pre-existing comorbidity, n (%)**				
HTN	38 (41.3%)	20 (43.5%)	18 (39.1%)	0.832
DM	19 (20.7%)	9 (19.6%)	10 (21.7%)	1.000
Heart disease	18 (19.6%)	8 (17.4%)	10 (21.7%)	0.793
Chronic kidney disease	0 (0.0%)	0 (0.0%)	0 (0.0%)	1.000
Respiratory disease	0 (0.0%)	0 (0.0%)	0 (0.0%)	1.000
Stroke	4 (4.3%)	3 (6.5%)	1 (2.2%)	0.617
Dyslipidemia	7 (7.6%)	3 (6.5%)	4 (8.7%)	1.000
**Mechanical CPR**	27 (29.3%)	13 (28.3%)	14 (30.4%)	1.000
**Post-cardiac arrest care**				
PCI	76 (82.6%)	38 (82.6%)	38 (82.6%)	1.000
**Outcomes at hospital discharge**				
Survival	27 (29.3%)	15 (32.6%)	12 (26.1%)	0.647
Good neurological outcome	16 (17.4%)	9 (19.6%)	7 (15.2%)	0.783

Abbreviations: TTM, targeted temperature management; IQR, interquartile range; CPR, cardiopulmonary resuscitation; EMS, emergency medical service; ECPR, extracorporeal cardiopulmonary resuscitation; ROSC, return of spontaneous circulation; ER; emergency room; DM, diabetes mellitus; HTN, hypertension; PCI, percutaneous coronary intervention...

[a] Any non-sustained ROSC event prior to ECPR

Table S6. Characteristics of the study population according to administration of TTM after propensity score in subgroup had bystander CPR.

Variables	Total (N=60)	ECPR without TTM (N=30)	ECPR with TTM (N=30)	p
**Age, years [median (IQR)]**	49.0 [41.5;61.5]	49.0 [36.0;58.0]	48.0 [45.0;62.0]	0.631
**Sex, male, n (%)**	50 (83.3%)	26 (86.7%)	24 (80.0%)	1.000
**Location of cardiac arrest**				
Public places	18 (30.0%)	9 (30.0%)	9 (30.0%)	1.000
**First cardiac rhythm at EMS**				
Shockable rhythms, n (%)	22 (36.7%)	10 (33.3%)	12 (40.0%)	0.928
Non-shockable rhythms, n (%)	6 (10.0%)	3 (10.0%)	3 (10.0%)	
Unknown	32 (53.3%)	17 (56.7%)	15 (50.0%)	
**EMS call to ECPR, mins***	30.0 [12.4-71.5]	3.9 [12.6-80.0]	41.0 [12.0-68.0]	0.663
**Non-sustained ROSC event** [a]	43 (71.7%)	22 (73.3%)	21 (70.0%)	1.000
**Pre-existing comorbidity, n (%)**				
HTN	17 (28.3%)	7 (23.3%)	10 (33.3%)	0.567
DM	12 (20.0%)	8 (26.7%)	4 (13.3%)	0.333
Heart disease	5 (8.3%)	3 (10.0%)	2 (6.7%)	1.000
Chronic kidney disease	0 (0.0%)	0 (0.0%)	0 (0.0%)	1.000
Respiratory disease	0 (0.0%)	0 (0.0%)	0 (0.0%)	1.000
Stroke	0 (0.0%)	0 (0.0%)	0 (0.0%)	1.000
Dyslipidemia	1 (1.7%)	0 (0.0%)	1 (3.3%)	1.000
**Mechanical CPR**	16 (26.7%)	8 (26.7%)	8 (26.7%)	1.000
**Post-cardiac arrest care**				
PCI	48 (80.0%)	24 (80.0%)	24 (80.0%)	1.000
**Outcomes at hospital discharge**				
Survival	16 (26.7%)	7 (23.3%)	9 (30.0%)	0.770
Good neurological outcome	9 (15.0%)	6 (20.0%)	3 (10.0%)	0.472

Abbreviations: TTM, targeted temperature management; IQR, interquartile range; CPR, cardiopulmonary resuscitation; EMS, emergency medical service; ECPR, extracorporeal cardiopulmonary resuscitation; ROSC, return of spontaneous circulation; ER; emergency room; DM, diabetes mellitus; HTN, hypertension; PCI, percutaneous coronary intervention..

* Time from EMS call to EMMO pump-on at ED

[a] Any non-sustained ROSC event prior to ECPR

Table S7. Characteristics of the study population according to administration of TTM after propensity score matching in subgroup had non-sustained ROSC event prior to ECPR.

Variables	Total (N=86)	ECPR without TTM (N=43)	ECPR with TTM (N=43)	p
**Age, years [median (IQR)]**	50.5 [41.0;63.0]	51.0 [41.0;61.5]	50.0 [41.5;63.5]	0.789
**Sex, male, n (%)**	76 (88.4%)	37 (86.0%)	39 (90.7%)	0.737
**Bystander CPR**	43 (50.0%)	21 (48.8%)	22 (51.2%)	1.000
**Location of cardiac arrest**				
Public places	21 (24.4%)	12 (27.9%)	9 (20.9%)	0.616
**First cardiac rhythm at EMS**				
Shockable rhythms, n (%)	27 (31.4%)	11 (25.6%)	16 (37.2%)	0.274
Non-shockable rhythms, n (%)	22 (25.6%)	14 (32.6%)	8 (18.6%)	
Unknown	37 (43.0%)	18 (41.9%)	19 (44.2%)	
**EMS call to ECPR, mins***	44.5 [12.6-74.0]	44.0 [12.6-80.0]	44.0 [12.7-68.0]	0.993
**Pre-existing comorbidity, n (%)**				
HTN	31 (36.0%)	16 (37.2%)	15 (34.9%)	1.000
DM	19 (22.1%)	11 (25.6%)	8 (18.6%)	0.603
Heart disease	17 (19.8%)	9 (20.9%)	8 (18.6%)	1.000
Chronic kidney disease	2 (2.3%)	1 (2.3%)	1 (2.3%)	1.000
Respiratory disease	0 (0.0%)	0 (0.0%)	0 (0.0%)	1.000
Stroke	0 (0.0%)	0 (0.0%)	0 (0.0%)	1.000
Dyslipidemia	5 (5.8%)	3 (7.0%)	2 (4.7%)	1.000
**Mechanical CPR**	25 (29.1%)	12 (27.9%)	13 (30.2%)	1.000
**Post-cardiac arrest care**				
Reperfusion treatment [b]	72 (83.7%)	36 (83.7%)	36 (83.7%)	1.000
**Outcomes at hospital discharge**				
Survival	24 (27.9%)	14 (32.6%)	10 (23.3%)	0.471
Good neurological outcome	17 (19.8%)	12 (27.9%)	5 (11.6%)	0.104

Abbreviations: TTM, targeted temperature management; IQR, interquartile range; CPR, cardiopulmonary resuscitation; EMS, emergency medical service; ECPR, extracorporeal cardiopulmonary resuscitation; ROSC, return of spontaneous circulation; ER; emergency room; DM, diabetes mellitus; HTN, hypertension; PCI, percutaneous coronary intervention.

* Time from EMS call to EMMO pump-on at ED
